# The effects of electroacupuncture and laser acupuncture therapy for patients with major trauma: A study protocol

**DOI:** 10.1097/MD.0000000000028367

**Published:** 2021-12-30

**Authors:** Chun-Ting Liu, Ting-Min Hsieh, Fu-Yuan Shih, Wei-Hung Lai, Ching-Hua Hsieh, Bei-Yu Wu, Yung-Hsiang Chen

**Affiliations:** a Department of Chinese Medicine, Kaohsiung Chang Gung Memorial Hospital and Chang Gung University College of Medicine, Kaohsiung, Taiwan; b Graduate Institute of Integrated Medicine, College of Chinese Medicine, Research Center for Chinese Medicine & Acupuncture, China Medical University, Taichung, Taiwan; c Department of Chinese Medicine, Dali Branch, Jen-Ai Hospital, Taichung, Taiwan; d Division of Trauma Surgery, Kaohsiung Chang Gung Memorial Hospital and Chang Gung University College of Medicine, Kaohsiung, Taiwan; e Department of Neurosurgery, Kaohsiung Chang Gung Memorial Hospital and Chang Gung University College of Medicine, Kaohsiung, Taiwan; f Division of Plastic and Reconstructive Surgery, Department of Surgery, Kaohsiung Chang Gung Memorial Hospital and Chang Gung University College of Medicine, Kaohsiung, Taiwan; g Fooyin University College of Nursing, Kaohsiung, Taiwan; h Department of Psychology, College of Medical and Health Science, Asia University, Taichung, Taiwan.

**Keywords:** acupuncture, cholinergic anti-inflammatory pathway, inflammation, laser acupuncture, major trauma

## Abstract

**Background:**

Major trauma is the leading cause of death in the young population. The inflammatory and anti-inflammatory responses are associated with posttraumatic morbidity and mortality; however, it is not fully clear how to reestablish the homeostasis in patients with major trauma.

**Methods:**

This study will be a prospective, randomized, placebo-controlled, partially double-blinded, three-armed trial. One hundred eighty participants diagnosed with major trauma will be randomly assigned to an electroacupuncture (EA), a laser acupuncture (LA), or a sham laser acupuncture group in a 1:1:1 ratio. All participants will undergo EA, LA, or sham laser acupuncture intervention once a day on 5 acupoints (LI4, PC6, ST36, SP6, and EX-HN1) for 14 consecutive days after enrollment. The primary outcome measure will be the length of hospital stay. Secondary outcomes will be inflammatory mediators, including serum C-reactive protein, interleukin (IL)-6, tumor necrosis factor-α, IL-1β, and IL-10. Clinical outcomes will be numeric rating scale scores for pain, sequential organ failure assessment, ICU length of stay, 30-day mortality, and WHO Disability Assessment Schedule. Data will be analyzed by *chi*-square test or *t* test for pairwise comparisons, as well as one-way ANOVA followed by post hoc Tukey method between groups.

**Objectives:**

The aim of this protocol is to investigate the clinical effects of EA and LA on major trauma.

**Trial registration:**

ClinicalTrials.gov Identifier: NCT04970433. Registered on July 21, 2021.

## 1 Introduction

Trauma remains the leading cause of death worldwide in patients under 45 years of age.^[1]^ Major trauma is defined as 1 or multiple severe life or limb-threatening injuries, or an Injury Severity Score (ISS) greater than 15.^[2]^ The comprehensive care of major trauma patients is a great challenge for physicians because of their unique characteristics, for sometimes surgical care is required beyond the restoration of the injured anatomy.^[3]^ The “two hit theory of injury” is that the host defense responses during major trauma include responses to both primary insult and secondary insult.^[4]^ After physicians initially manage the trauma-related tissue injury and stabilize the vital signs, ischemia/reperfusion injury and hyperinflammation in the secondary hit will induce immunological responses such as postinjury multiple organ dysfunction syndrome and multiple organ failure, which can negatively affect the preservation of life.[^3^,^4^] Accounting for the influence of systemic posttraumatic inflammatory and metabolic reactions is important to reduce posttraumatic morbidity and mortality; however, it is not fully clear how to reestablish the homeostasis in major trauma patients.

After major trauma, patients suffer inflammatory response syndrome, which manifests as elevated serum levels of C-reactive protein and proinflammatory cytokines such as tumor necrosis factor (TNF)-α, interleukin (IL)-1β and IL-6.^[5]^ IL-6 has been correlated with ISS scores, incidence of multiple organ dysfunction syndrome, sepsis and survival prognosis.^[5]^ Following the hyperinflammatory stage is the compensatory anti-inflammatory response syndrome, which is characterized by production of anti-inflammatory cytokines such as IL-10.^[6]^ The balance between pro- and anti-inflammatory mediators could influence survival rates.[^6^,^7^] Many diseases that include major trauma followed by pain and inflammatory response syndrome may cause a severe physiologic stress response, which is associated with the autonomic nervous system. The autonomic nervous system comprises both the sympathetic nervous system (SNS) and the parasympathetic nervous system (PNS). The SNS cooperates with the PNS in regulating the protection of the body against injury and stress. Hyperactivity of the SNS may be an adaptive response to ensure that damaged tissues receive adequate oxygen. However, in severe trauma, this hyperadrenergic state can be maladaptive, resulting in damage to the myocardium and other critical organs. It has been reported that vagus nerve stimulation can exhibit anti-inflammatory effects via the cholinergic-anti-inflammatory pathway.[^8^,^9^] The interaction between the central nervous system and peripheral immune response, the so-called cholinergic-anti-inflammatory pathway, plays an important role in the control of inflammation.

Acupuncture therapy is currently widely applied in the treatment of many diseases. ST-36 (Zusanli) is used in various inflammatory diseases and seems to have broad spectrum anti-inflammation effects.^[10]^ The cholinergic anti-inflammatory pathway could provide a possible physiological mechanism for the reported anti-inflammatory actions of acupuncture, which are mediated by the down-regulation of IL-6 and IL-1β.^[11]^ In the literature, the ST-36 and PC-6 (Neiguan) acupoints are reported to significantly increase vagal activity and decrease TNF-α and IL-6 levels in patients with multiple trauma.^[12]^ LI4 (Hegu) and SP6 are reported to increase vagal activity in dysmenorrhea treatment.^[13]^ In addition, EX-HN1 (Sishencong) points appear to enhance PNS activities and suppress SNS activities in humans.^[14]^ We hypothesize that acupuncture may modulate the autonomic nervous system to balance pro- and anti-inflammatory mediators in patients with major trauma, which in turn may contribute to reductions in morbidity and mortality. Hence, we have designed a prospective clinical trial to investigate the effects of acupuncture in patients with major trauma.

## 2 Methods/design

### 2.1 Ethics approval

This protocol has been reviewed and approved by the Human Ethics Committee of the Chang Gung Medical Foundation Institutional Review Board (IRB no. 202002416A3; version 1 on March 29, 2021). The protocol identification number at https://clinicaltrials.gov is NCT04970433. This study will be conducted in accordance with the principles of the Declaration of Helsinki. Before participation, both verbal and written forms of detailed information about the trial will be provided by trauma surgery physicians at Kaohsiung Chang Gung Memorial Hospital. All participants will provide voluntarily signed informed consent that has been approved by the ethics committee prior to enrollment. Personal information about potential and enrolled participants will be collected, shared, and maintained in an independent and secure storage space to protect the participants’ confidentiality before, during, and after the trial. We will present the final results and submit them for publication in peer-reviewed journals. Chun-Ting Liu will have access to the final trial dataset and disclosure of contractual agreements that limit such access for investigators.

### 2.2 Study design

**Figure 1:**
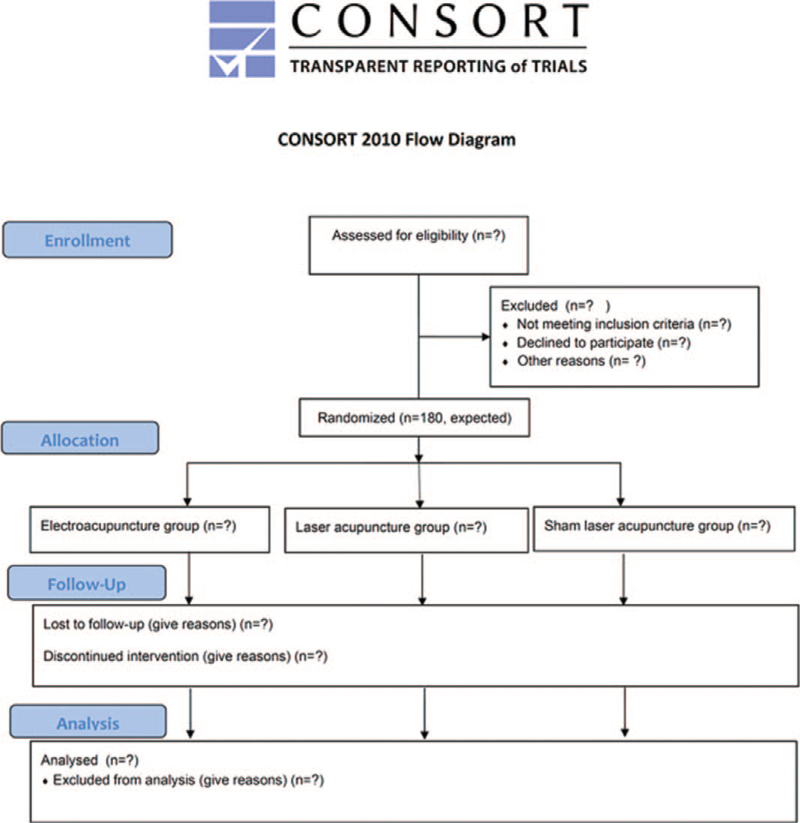
The flowchart of the trial.

This prospective, randomized, placebo-controlled, partially double-blinded, three-armed trial began at the Kaohsiung Chang Gung Memorial Hospital in Taiwan in August 2021 and will continue until July 2024. One hundred eighty participants will be randomly assigned to the electroacupuncture group (EA, n = 60), laser acupuncture group (LA, n = 60) or control group (sham laser acupuncture [SLA], n = 60). All participants will receive EA, LA, or SLA after enrollment, once daily for 14 days, during hospitalization. The study design, which is based on the Consolidated Standards of Reporting Trials (CONSORT) 2010, is depicted in Figure 1.

### 2.3 Participants

The information on the trial will be provided by trauma surgery physicians at Kaohsiung Chang Gung Memorial Hospital. Participants’ eligibility for the study will be assessed by trauma surgeons. Written informed consent will be obtained from all participating patients before randomization. All patients with major trauma will be managed from the time of admission by the trauma surgery team, who will have no information about the allocation results.

The study inclusion criteria are as follows: patients aged 20 years or older; patients diagnosed with major trauma with ISS ≥16; consent to participate in the study. Participants with the following conditions will be excluded: pregnancy; malignancy; pacemaker; status epilepticus; severe medical disease, for example severe congestive heart failure, cirrhosis, and end stage renal disease; life threatening condition; prior history of drug or alcohol dependence; immunodeficiency; vagotomy. Participants will be excluded from the trial under the following conditions: unstable vital signs and/or need for first aid during study period; deterioration of clinical condition and determination of unsuitability to continue this study by medical staff; voluntary decision by participants to withdraw from the trial at any time. Participants will be dropped from the trial under the following conditions: unstable vital signs and need for first aid during study period; deterioration in clinical condition leading to an assessment by medical staff of unsuitability to continue the study; participant's decision to withdraw from the trial at any time.

### 2.4 Sample size, blinding, and randomization

No related studies on the effect of inflammatory mediators and clinical outcomes of acupuncture in patients with major trauma have been conducted. However, TNF-α is well known to play a major pro-inflammatory role in local and systemic inflammatory response, including major trauma. Thus, we determined the necessary sample size based on a previous study of the effects of acupuncture on inflammatory responses in septic patients via the change of TNF-α.^[15]^ The TNF-α levels on day 7 were 20.32 ± 11.30 in the experimental group and 32.99 ± 20.62 in the control group. Anticipating a power of 95% (1-β = 0.95), statistical significance (α = 0.05) of 95% and a dropout rate of 20% to 25%, a sample size of 60 participants was estimated for each group by G∗Power analysis, for a total of 180 participants in this trial.

Recruited patients will be randomized in a ratio of 1:1:1 into 3 parallel treatment groups of 60 participants: EA, LA, and SLA. All the participants will be told that they will receive one kind of acupuncture treatment in addition to the conventional treatment. The participants will not be notified about their group allocation or the kind of acupuncture treatment before randomization. The participants will be randomly assigned to the 3 groups after providing written informed consent. Randomization will be performed with a 1:1:1 allocation using a computer-generated sequence executed by an independent researcher, who will not be involved in the inclusion or exclusion process, treatment, or assessment procedures. Participants receiving EA will not be blinded to EA in this trial. Participants receiving LA or SLA will be blinded to the LA. The outcome assessors and data analysts will be blinded to the allocation of interventions by using labels A, B, and C for the 3 groups until the trial is completed.

### 2.5 Interventions

The same acupoint regimen, bilateral LI4, PC6, ST36, SP6, and EX-HN1, which are localized according to the WHO Standardized Acupuncture Point Location guidelines, will be applied in each group. Participants will be treated with EA, LA, or SLA once daily for up to 14 days during hospitalization. Certified traditional Chinese medicine physicians with at least 5 years of experience in acupuncture will perform the treatment, and they will not participate in the randomization, outcome measurement, or statistical analysis.

#### 2.5.1 Intervention group/electroacupuncture group

The skin areas of the acupoints will be sterilized by a wipe with 75% ethanol. Disposable, sterilized, stainless steel needles will be inserted to a depth of 15 to 35 mm. All needles will be rotated manually in combination with the lifting–thrusting method to elicit needle sensation (De qi). Electrical stimulation will then be applied using an EA apparatus. For this stimulation, a pair of electrodes connecting acupoints LI4 with PC6 and another pair of electrodes connecting ST36 with SP6 will be used. EA stimulation will last for 15 minutes with a continuous wave of 2 Hz and a current intensity of 0.1 to 1 mA. The current intensity will be increased until the skin around the acupoints shivers.

#### 2.5.2 Intervention group/laser acupuncture group

Participants allocated to the LA group will receive LA therapy at the same acupoints used in the EA group. The LA therapy will be performed with the gallium aluminum arsenide LaserPen (maximal power, 150 mW; wavelength, 810 nm; area of probe, 0.03 cm^2^; power density, 5 W/cm^2^; and pulsed-wave; RJ-Laser, Reimers & Janssen GmbH, Waldkirch, Germany). The laser will be applied to each point for 40 seconds, which will deliver 3 J of energy at each of the acupoints.

#### 2.5.3 Control group/sham laser acupuncture group

Participants in the control group will receive SLA treatment without any laser output. The acupuncture points, application duration, and total number of treatments will be the same as those in the LA group.

### 2.6 Outcome measurements

The primary outcome measurement will be the length of hospital stay, which will be calculated from electronic health records. Secondary outcome measurements will consist of pro- and anti-inflammatory mediators as well as clinical outcomes associated with major trauma. Inflammatory mediators, namely, serum C-reactive protein, IL-6, TNF-α, IL-1β, and IL-10, and short-term clinical outcomes, namely, numeric rating scale score for average pain and sequential organ failure assessment (SOFA), will be measured within 48 hours and at 7 and 14 days post major trauma. In addition, ICU length of stay and 30-day mortality will be recorded. The 12-item version of the WHO Disability Assessment Schedule 2.0 (WHODAS 2.0) will be used to evaluate long term clinical outcomes at 3 and 6 months after the major trauma.

The numeric rating scale is a simple tool on which patients verbally indicate a score on a 10-point scale (0 = no pain and 10 = worst possible pain). The SOFA score numerically quantifies the number and severity of failed organs. The score is based on 6 different scores, 1 each for the respiratory, cardiovascular, hepatic, coagulation, renal, and neurological systems. The criteria for assessment of the SOFA are based on a previous study.^[16]^ The WHODAS 2.0 measures disability across 6 domains^[17]^: understanding and communication, getting around, self-care, getting along with others, life activities, and participation in society. This questionnaire contains 12 items rated on a Likert-type scale of 1 (no disability) to 5 (very severe disability). Patients will be evaluated for disability status with the brief WHODAS 2.0 12-item instrument by in-person interview with patients or their family members if the patient is not alert, or postdischarge by telephone interview at 3 and 6 months posttrauma.

### 2.7 Statistical analysis

Between-group comparisons of the EA and the LA group with SLA and pairwise comparisons of the EA and LA groups will be performed. Quantitative variables, presented as mean ± SD, will be analyzed by the Student *t* test or one-way ANOVA followed by post hoc Tukey method. Qualitative variables, expressed as a number (percentage), will be analyzed using the chi-square test. All analyses will be performed with SPSS software. All analyses will be performed with SPSS 22.0 for Windows (Statistics 22.0, SPSS, IBM, New York, NY). Differences will be considered statistically significant at *P* < .05.

### 2.8 Data monitoring

No data monitoring committee is needed because acupuncture and LA are generally practiced and minimally invasive interventions. However, possible adverse events related to EA are hematoma, pain, nerve irritation, infection, and tiredness, and the only relevant adverse event related to LA therapy is accidental irradiation of the eyes.

## 3 Discussion

Although the research on acupuncture has grown markedly, appropriate designs for clinical acupuncture trials remain a methodological challenge. The most difficult issues are the design of the control group and implementation of the principle of “double-blinding.” The commonly used controls in acupuncture trials include non-intervention control, non-insertion sham control, and needle-insertion at sham or real acupoints.^[18]^ However, the blinding effect of non-insertion sham control may not last for a long time, and any needle-insertion control may have a non-specific effect, such as unspecific endorphin release.[^18^,^19^] Accordingly, no reliable blinding methodology for needle acupuncture has been achieved so far. Recently, SLA has been used as a control for needle acupuncture trials. SLA reportedly can serve as a valid placebo control in LA studies due to their similar credibility and the lack of sensory input on the peripheral nervous system, and it can serve as a sham control for acupuncture trials when there is a need to evaluate the effects of needling per se.^[20]^ Currently, various acupuncture modalities, such as EA and manual acupuncture, are available to clinicians. Although EA and manual acupuncture may have different therapeutic effects via different mechanisms, EA seems to have a faster and better analgesic effect than that of manual acupuncture.^[21]^ In this trial, a three-armed design will be applied to investigate the effects of EA and LA for comparison with SLA in neuroimmune modulation and clinical outcomes for patients with major trauma. With this design, SLA treatment meets all requirements to produce the same non-specific effects as LA treatment, which can verify the efficacy of the selected acupoints. SLA can serve as a sham procedure and LA can serve as an active comparator for evaluation of the needling effect. Although the needling effect may be underestimated, we think this is an appropriate design for an acupuncture trial.

Major trauma is a major cause of death or long-term disability in the young population worldwide. Such injuries may cause the breakdown of families and impose heavy burdens on the country and society. Saving the lives of these patients and reducing their disability benefits both society and the economy. We believe that it is important to seek less invasive treatments for many diseases, including major trauma. Vagus nerve stimulation has been applied diseases such as refractory seizure, treatment-resistant depression, and migraine, and emerging research is expanding the potential use of vagus nerve stimulation in cardiac diseases, neurological disorder, and rheumatoid arthritis.[^22^,^23^,^24^,^25^] Acupuncture is reported to increase the activity of the vagus nerve and is not as invasive as vagus nerve stimulation devices. For this study, we have selected the acupoints LI4, PC6, ST36, SP6, and EX-HN1 to examine the effects and possible mechanisms associated with neuroimmune modulation in patients with major trauma. In addition, short-term outcomes, including ICU stay, hospital stay and 30-day mortality, and long-term outcomes, including 3- and 6-month disability, will be measured to examine the relationship of acupuncture to inflammatory mediators and clinical significance. To the best of our knowledge, no similar designs or related studies have been published to date.

In conclusion, the results of this study are expected to indicate the effects of acupuncture as an alternative treatment in the management of major trauma, in addition to conventional treatments.

## Acknowledgments

The authors sincerely thank all the teachers at the Graduate Institute of Integrated Medicine, China Medical University, for statistic work. Ting-Min Hsieh and Yung-Hsiang Chen contributed equally to this study.

## Author contributions

**Conceptualization:** Chun-Ting Liu, Ting-Min Hsieh.

**Data curation:** Bei-Yu Wu.

**Formal analysis:** Bei-Yu Wu.

**Investigation:** Fu-Yuan Shih, Wei-Hung Lai.

**Project administration:** Chun-Ting Liu, Fu-Yuan Shih, Wei-Hung Lai.

**Supervision:** Ching-Hua Hsieh.

**Writing – original draft:** Chun-Ting Liu.

**Writing – review & editing:** Ting-Min Hsieh, Yung-Hsiang Chen.
